# Associations of hyperglycemic emergency and severe hypoglycemia incidences with seasonality and ambient temperature among pregnant women with diabetes: a nested case-control study in Taiwan

**DOI:** 10.1265/ehpm.22-00003

**Published:** 2022-03-12

**Authors:** Wen-Hsuan Hou, Jia-Ling Wu, Chin-Li Lu, Lilis Sulistyorini, Muhammad Atoillah Isfandiari, Chang-Ta Chiu, Chung-Yi Li

**Affiliations:** 1 Department of Physical Medicine and Rehabilitation, Taipei Medical University Hospital, Taipei, Taiwan; 2 Master Program in Long-Term Care and School of Gerontology Health Management, College of Nursing, Taipei Medical University, Taipei, Taiwan; 3 Department of Geriatrics and Gerontology, Taipei Medical University Hospital, Taipei, Taiwan; 4 Graduate Institute of Clinical Medicine, College of Medicine, Taipei Medical University, Taipei, Taiwan; 5 Center of Evidence-Based Medicine, Department of Education, Taipei Medical University Hospital, Taipei, Taiwan; 6 Department of Public Health, College of Medicine, National Cheng Kung University, Tainan, Taiwan; 7 Graduate Institute of Food Safety, College of Agriculture and Natural Resources, National Chung Hsing University, Taichung, Taiwan; 8 Department of Environmental Health, Faculty of Public Health, Universitas Airlangga, Surabaya, Indonesia; 9 Department of Epidemiology, Faculty of Public Health, Universitas Airlangga, Surabaya, Indonesia; 10 Department of Dentistry, An Nan Hospital, China Medical University, Tainan, Taiwan; 11 Department of Public Health, College of Public Health, China Medical University, Taichung, Taiwan; 12 Department of Healthcare Administration, College of Medical and Health Science, Asia University, Taichung City, Taiwan

**Keywords:** Seasonal variation, Temperature, Pregnancy in diabetes, Pregnancy complications, Case-control studies

## Abstract

**Background:**

Associations of acute glycemic complications with season and ambient temperature have been reported in general population with diabetes. However, little is known about the risks of acute glycemic complications in relation to season and ambient temperature in pregnant women, who are likely to be even more vulnerable. This work aimed to investigate the associations of season and ambient temperature with pregnancies complicated with hyperglycemia emergency or severe hypoglycemia.

**Methods:**

Two separate case-control studies were nested within 150,153 pregnancies by women with type 1, type 2, or gestational diabetes between 2009 and 2014 in Taiwan. Hyperglycemia emergency (mainly diabetic ketoacidosis and hyperosmolar hyperglycemic state) and severe hypoglycemia occurred in 77 and 153 diabetic pregnancies (cases), respectively. Ten control pregnancies were randomly selected for each case by matching each case pregnancy on type of diabetes (i.e., T1DM, T2DM, or GDM), maternal age on the date of acute glycemic complication occurrence (i.e., index date), and “length of gestation at risk” (i.e., period between conception and index date). Meteorological parameters were retrieved from 542 meteorological monitoring stations across Taiwan during 2008–2014. Conditional logistic regression analysis with generalized estimation equation was separately performed to estimate the covariate adjusted odds ratios (ORs) of each of the two acute glycemic complications in association with season and ambient temperature within 30 days prior to the index date.

**Results:**

Compared to summer, winter season was associated with a significantly elevated risk of severe hypoglycemia with an OR of 1.74 (95% confidence interval (CI) 1.08–2.79). The OR of hyperglycemic emergency was also elevated in winter season at OR of 1.88, but the significance is only marginal (95% CI 0.97–3.64, *p* = 0.0598). Subgroup analyses further noted that such seasonal variation was also observed in pregnancies with pre-pregnancy type 1 diabetes and gestational diabetes. On the other hand, ambient temperature was not significantly associated with the two acute glycemic complications.

**Conclusions:**

A moderately but significantly elevated risk of severe hypoglycemia was found in pregnant women with diabetes during winter season, and such increased risk was more evident in pregnancies with T1DM.

**Supplementary information:**

The online version contains supplementary material available at https://doi.org/10.1265/ehpm.22-00003.

## Graphical Abstract

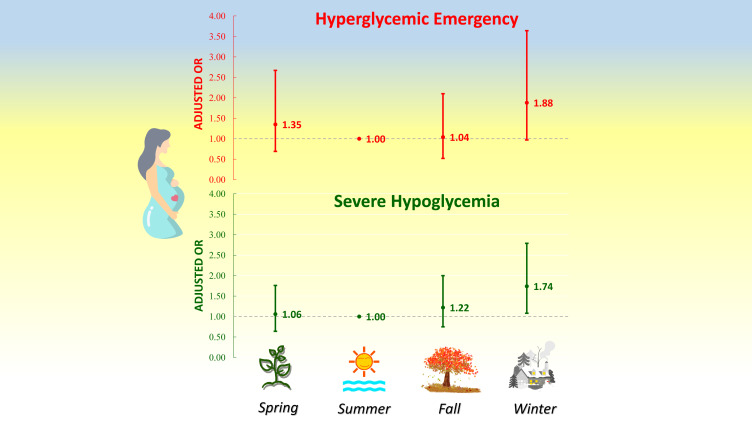

## Introduction

Diabetic ketoacidosis (DKA) and hyperosmolar hyperglycemic state (HHS) are two severe hyperglycemia emergencies of diabetes, which, if during pregnancy, may compromise the fetus and the mother and is associated with high fetal and maternal mortality [1]. DKA during pregnancy is extremely rare with a reported incidence between 0.5% and 3% of all diabetic gestations [2]. HHS is a life-threatening emergency that is less common but has higher mortality rate than its counterpart, DKA. Mortality rates for HHS approach 20% in the general population [3], and infections and a history of insulin therapy omission are the most common causes of DKA in these patients [2].

Severe hypoglycemia is another acute glycemic complication that is common during pregnancy with diabetes. One study reported that 23% of women with type 1 diabetes mellitus (T1DM) have a severe hypoglycemic attack at least once during pregnancy, and many experienced several episodes [4]. Severe hypoglycemic attacks may result in consciousnesses loss, convulsion, and even fall or trauma, thus causing harms to mothers and offspring [1]. Hypoglycemia during pregnancy is associated with several risk factors, including being in the first trimester, having had hypoglycemic attacks prior to pregnancy, being malnourished, and experiencing appetite loss without adequate or regular food intake [5].

In addition to the abovementioned clinical risk factors for the above two acute glycemic events, the potential influence of ambient temperature on blood sugar levels has been of concern. In countries near the equator, the hot climate is assumed to be the cause of many DKA cases because patients with diabetes sweat profusely and become dehydrated, leading to the onset of hyperglycemia [6]. Concerning the temperature and hypoglycemia relationship, a German study found the frequency of severe hypoglycemia remarkably increases at temperatures above 20 °C (+18%) and below 10 °C (+15%) compared with mild temperatures (10 °C–20 °C) [7]. A recent Taiwanese study noted a seasonal variation in the incidence rate of hypoglycemia, with a higher number of cases during the cold weather [8].

While most studies on season or ambient temperature in relation to acute glycemic complications were performed in the general diabetes population, little information is available for pregnant women with diabetes [9]. Women tend to become insulin resistant as their pregnancy progresses. Along with hormonal changes, pregnancy can impede the glucose regulation of women [1, 4]. This population-based study was conducted to investigate the risk of acute glycemic events in association with season and ambient temperature in pregnant women with diabetes.

## Materials and methods

The study proposal was approved by the Institutional Review Board of National Cheng Kung University Hospital (No. B-ER-107-014).

### Setting and sources of health data

Taiwan is an island with 394 kilometers long and 144 kilometers wide at its broadest point, and includes a number of smaller islands. Foothills and mountains cover over two thirds of the island. The daily temperatures range between 17–24 °C in November, dropping to 12–19 °C in January and then rising to 14–22 °C in March. The highest daily temperature was seen in July and August at 24–35 °C. (https://www.countryreports.org/country/Taiwan/geography.htm#:~:text=The%20daily%20temperatures%20range%20between,especially%20in%20the%20mountainous%20areas.)

Data analyzed comprised several population-based registries, including Birth Notifications (2009–2014), Household Registration (2009–2014), and the National Health Insurance (NHI) program claims data (2006–2014). The Birth Notifications include various sociodemographic and pregnancy-related variables including maternal age and married status, maternal education, offspring gender, fetal death, maternal complications during pregnancy, gestational age, birth weight, congenital abnormalities, and Apgar scores. Taiwan’s Birth Notifications have been considered valid and complete [10].

The Household Registration provides information on aboriginality and low-income family status. The NHI dataset, a reimbursement-based medical claim registry, includes information on maternal co-morbidity during pregnancy. The NHI program is supervised by the National Health Insurance Administration (NHIA), Ministry of Health and Welfare. The program covers more than 99% of Taiwan’s residents, and reimbursement are performed for nearly all inpatient and outpatient visits. The NHIA performs random sampling on claim data for expert reviews to ensure the accuracy of medical claims data [11, 12]. Information on co-morbidity was retrieved based on all outpatient/inpatient visits by mothers in the 3-year period prior to pregnancy.

### Research design and study sample

Two nested case-control studies which shared the same methodology were conducted to separately assess the association of season / ambient temperature with hyperglycemia emergency and severe hypoglycemia, respectively. A total of 1,212,569 pregnancies by 938,992 mothers were registered in Birth Notifications between 2009 and 2014. Information on the socio-demographic characteristics and season of delivery for all 1,212,569 pregnancies was presented in Supplementary Table 1. The following pregnancies were excluded: pregnancies with missing mother’s personal identification number (*n* = 9), missing mother’s age (*n* = 9,350) or mothers aged <15 or >50 years (*n* = 291), gestational age <20 or >42 weeks, multiple births (*n* = 19,024), and non-diabetic pregnancies (*n* = 1,019,996). A total of 150,153 diabetic pregnancies (i.e., type 1, type 2, or gestational diabetes [GDM]) were finally included in the sample. Among them, 1,366 pregnancies were by mothers with T1DM (ICD-9CM: 250.x1 or 250.x3), 8,681 by mothers with type 2 diabetes mellitus (T2DM) (ICD-9CM: 250.x0 or 250.x2), and 140,106 by mothers with GDM (ICD-9CM: 648.0 or 648.8).

The case and control series were nested within the 150,153 cohort of singleton diabetic pregnancies. In the hyperglycemia emergency series, the initial cases were 79 pregnancies, including 73 with DKA (ICD-9-CM: 250.1) and 6 with HHS (ICD-9-CM: 250.2). The index pregnancies accompanied by the following diseases at the same visit for hyperglycemia emergency were further excluded since these diseases are known to trigger incidence of DKA and HHS: acute myocardial infarction, ischemic heart disease, coronary revascularization procedures, non-traumatic hemorrhagic stroke, ischemic stroke, acute pancreatitis, urinary tract infection, acute respiratory infections, pneumonia and influenza, tuberculosis, sepsis, acute renal failure, acute respiratory failure, hepatic failure, and disseminated intravascular coagulopathy syndrome [13]. Finally, 77 pregnancies (by 61 mothers) with hyperglycemia emergency, including 53 with T1DM, 3 with T2DM, and 21 with GDM in mothers, were included.

**Fig. 1:**
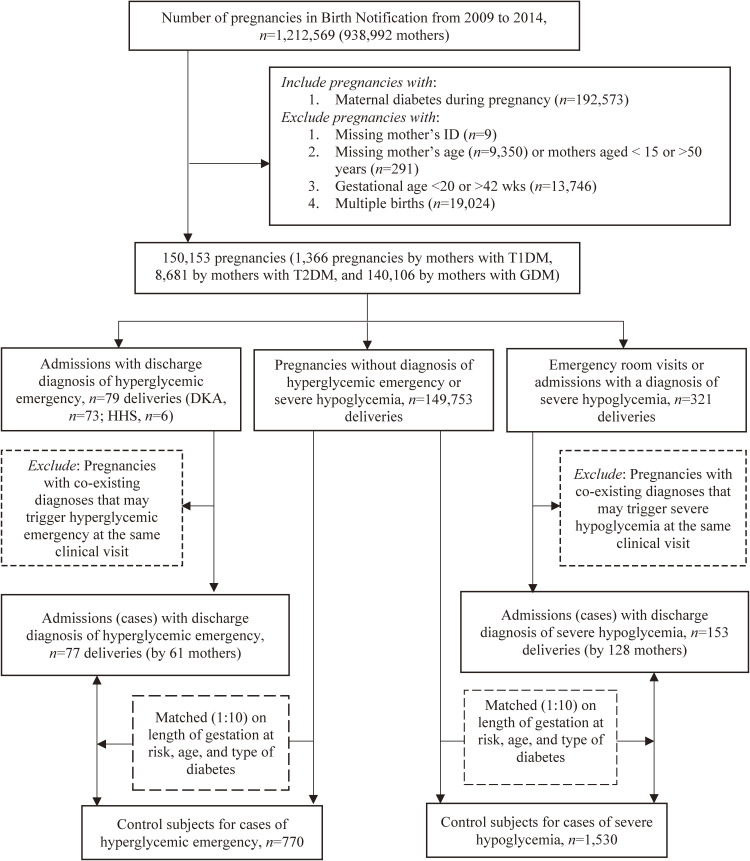
Flow chart of enrolling cases and controls.

The American Diabetes Association [14] defines severe hypoglycemia as an event that requires the assistance of another person. However, our study was based on claim data, which might have missed some cases who did not seek medical cares. In the severe hypoglycemia series, the initial cases included only 321 pregnancies with emergency room visits or admissions for severe hypoglycemia. After the modification of the coding algorithm proposed by Ginde et al. [15], the following ICD-9-CM codes were considered as severe hypoglycemia: 250.8, 251.0, 251.1, 251.2, 270.3, and 962.3. Cases with co-existing diagnoses of kidney disease, cardiovascular disease, heart failure, or depression at discharge were excluded. A total of 153 pregnancies (by 128 mothers), including 69 with T1DM, 13 with T2DM, and 71 with GDM in mothers, formed the final case series of severe hypoglycemia. Fig. 1 shows the flowchart of sample enrollment.

Conception date was determined based on the date of end-of-pregnancy and gestational age. The period between the date of conception and date of hyperglycemia emergency / severe hypoglycemia incidence (i.e., index date) was considered as “length of gestation at risk” and calculated for each case. In our sample, 9 pregnancies suffered from both hyperglycemia emergency and severe hypoglycemia during the same pregnancy, accounting for 11.7% (9/77) and 5.9% (9/153) of hyperglycemia emergency and severe hypoglycemia cases, respectively. The median interval between two acute glycemic complications in the same pregnancy was 58 days, with an interquartile range of 81 days, which was considered appropriate to analyze these two end-points separately.

Control pregnancies were selected stepwise using individual matching technique. For each index case pregnancy, all non-case pregnancies that had a gestational age longer than the “length of gestation at risk” of index case pregnancy were first selected. The “length of gestation at risk” of the index case pregnancy was then assigned to all selected candidates of control pregnancies; and the last day of the “length of gestation at risk” was considered as index date for those potential control pregnancies to ensure that the cases and their potential controls had the same “length of gestation at risk.” Second, 10 control pregnancies were randomly selected by matching each index case pregnancy on type of diabetes (T1DM, T2DM, or GDM), maternal age on the index date, and “length of gestation at risk”. (Fig. 1) A control subject can only be selected once and can’t be a case if she later developed acute glycemic complications. The decision for a case/control ratio of 1:10 was made to achieve sufficient statistical power.

### Exposure assessment

Seasonality was defined as the following: spring: Mar. – May; summer: Jun. – Aug.; fall: Sep. – Nov.; and winter: Dec. – Feb. The index date was used to determine the season for both cases and controls.

Monitoring meteorological data were obtained from the 542 meteorological stations across Taiwan during 2008–2014. Ambient temperature was measured on an hourly basis in a day. All hourly measurements in a day were averaged to indicate daily average temperature (°C); the highest and lowest measurements in a day were considered as the daily maximum and minimum temperature (°C), respectively. The monitoring daily data also included temperature range (°C) (i.e., maximum minus minimum), relative humidity (%), sunshine duration (h/day), apparent temperature (°C), and precipitation (mm/day). Daily apparent temperature was also calculated from hourly measurements of ambient temperature, wind, humidity, and radiation from the sun [16].

Maternal residential city district/township at the time of end-of-pregnancy was used for exposure assessment. Meteorological data retrieved from the meteorological station(s) nearest to the center of residential city district/township were used to characterize exposure, which was based on a 30-day period prior to the index date. We decided to use the 30-day mean daily temperature as the exposure measure in this study to obtain a more stable estimate of temperature exposure [17, 18].

### Covariates

The maternal sociodemographic characteristics included age, aboriginality, educational level, urbanization level of residence, monthly-income-based NHI insurance premium, low-income family status, and marital status on index date. The classification scheme for determining urbanization level was proposed by Liu et al. [19], who categorized all 316 city districts and townships in Taiwan into seven clusters. In the present study, the most and second most urbanized clusters were regarded as “urban” and “satellite” areas, respectively, and the remaining ones were classified as “rural” areas [20, 21].

We also estimated the fine particulate matter (2.5 µm or less in diameter [PM_2.5_]) within 30 days before the index date. Previous studies reported associations of air pollution with metabolic syndrome and T2DM, probably through increased inflammation, oxidative stress, and endoplasmic reticulum stress [22, 23]. Data of PM_2.5_ were retrieved from 76 fixed-site air quality monitoring stations of Taiwan Air Quality Monitoring Network (http://taqm.epa.gov.tw/taqm/en/PsiMap.aspx). PM_2.5_ concentration was estimated for the center point coordinator of each of the 316 city districts and townships through spatial analysis (i.e., ordinary kriging) on ArcGIS Desktop (version 10 software; ESRI Inc., Redlands, CA, USA) [24]. The spatial interpolation and cross-validation approach interpolated the exposure concentration to a regular grid (250 × 250 m^2^) across Taiwan. Cross-validation was based on the pollutant data of the AQMSs within 3 km outside the city district and township boundaries [24].

30-day mean daily apparent temperature was also considered as covariates. The other meteorological parameters were not considered in the analysis mainly because apparent temperature is a composite indicator of ambient temperature, humidity, wind, and radiation from the sun.

Information on co-morbidity history was retrieved from the medical claims in 3 years prior to the index date. The selected co-morbidity was considered as potential risk factors for hyperglycemia emergency [14, 25] or severe hypoglycemia [26, 27]. The ICD-9-CM codes for the above co-morbidity are listed in Supplementary Table 2. Due to unavailability of the information on lifestyle related to glycemic conditions, such as smoking habit, alcohol drinking, and body mass index from our research data, we were unable to consider these potential confounders in this study.

### Statistical analysis

We first compared socio-demographic characteristics, co-morbidity, air pollutant concentration, and birth outcomes between cases and controls. Univariate and multivariate conditional logistic regression models were applied to calculate crude and adjusted odds ratios (ORs) as estimates of the relative risk and 95% confidence intervals (CIs), which were employed to measure the associations between season / 30-day mean daily average temperature and two study outcomes. Generalized estimation equation, which specifies an exchangeable structure of a working correlation matrix to consider the within-subject’s correlations [28, 29] was applied in the logistic regression models to account for the pregnancies by the same mothers. The GEE uses robust standard error estimates to consider within-subject correlations of the case status.

In addition to sociodemographic characteristics and air pollutants, only the selected co-morbidity with sufficient case numbers was considered in the multivariate model. Although prior histories of urinary tract infection, acute respiratory infections, and pneumonia were adjusted in the regression model for the analysis of hyperglycemia emergency, only kidney disease and depression were adjusted in the regression model for the analysis of severe hypoglycemia. Temperature effect was assessed by first treating temperature as a continuous variable [30]. Estimated slope coefficients and the standard errors of the mean were also examined, and no indication of collinearity between season, ambient temperature, and other temperature related covariates was found in the multivariate logistic regression models [31]. The mean (± standard deviation, SD) of 30-day ambient temperature prior to index date for spring, summer, fall, and winter was 24.91 (± 3.33), 29.00 (± 1.48), 26.64 (± 2.54), and 19.33 (± 2.80) °C. Although there is a seasonal variation in ambient temperature noted in our sample, such variation is not substantial in the sub-tropical region. All statistical analyses were performed using SAS statistical software (SAS System for Windows, Version 9.4, SAS Institute Inc., Cary, NC, USA), and the significance level was set at α < 0.05.

## Results

Hyperglycemia emergency occurred more frequently to indigenous, natively born, low educated, lower income, unmarried, and rural living mothers. The prevalence of most selected co-morbidity was low in both controls and hyperglycemia emergency cases. Pregnancies with hyperglycemia emergency had higher prevalence of urinary tract infection and pneumonia than those without. Similar discrepancies in maternal socio-demographic characteristics were found for cases and controls in the severe hypoglycemia series. With respect to co-morbidity, a higher percentage of severe hypoglycemia cases than controls had a history of severe hypoglycemia (Table 1). Little differences in concentration of PM_2.5_ was observed between cases and controls for the two series of case and control. Distributions of season were also similar between cases and controls in two series. Table 1 also shows birth outcomes between cases and controls for the two series. Women with hyperglycemia emergency or severe hypoglycemia had shorter mean gestational age and lower birth weight than the controls, mainly due to a shorter gestational age among case women.

**Table 1:** Characteristics of study subjects.

**Variables**	**Hyperglycemia emergency**		**Severe hypoglycemia**	

	**Cases (*n* = 77)**	**Controls (*n* = 770)**	**Cases (*n* = 153)**	**Controls (*n* = 1,530)**
	mean ± SD / *n* (%)	mean ± SD / *n* (%)	mean ± SD / *n* (%)	mean ± SD / *n* (%)
***Matching variables***				
Duration of gestation (days) prior to the index date (i.e., “gestation at risk”)	154.5 ± 65.83	154.5 ± 65.41	155.3 ± 83.31	155.3 ± 83.05
Age at the index date (years)	29.53 ± 5.99	29.53 ± 5.95	30.14 ± 4.85	30.14 ± 4.83
Type of diabetes				
Type 1	53 (68.83)	530 (68.83)	69 (45.10)	690 (45.10)
Type 2	3 (3.90)	30 (3.90)	13 (8.50)	130 (8.50)
GDM	21 (27.27)	210 (27.27)	71 (46.40)	710 (46.40)
***Maternal sociodemographic status***				
Maternal age at index date (years)				
15–24	18 (23.38)	180 (23.38)	24 (15.69)	240 (15.69)
25–29	19 (24.68)	190 (24.72)	36 (23.53)	360 (23.53)
30–34	20 (25.97)	203 (26.28)	63 (41.18)	631 (41.22)
35–50	20 (25.97)	197 (25.63)	30 (19.61)	299 (19.56)
Aboriginal people	16 (20.78)	61 (4.19)	7 (4.70)	55 (3.78)
Born in Taiwan	77 (100.00)	735 (95.45)	149 (97.39)	1,448 (94.62)
Education				
Junior high school or below	9 (11.84)	37 (4.82)	13 (8.72)	69 (4.49)
Senior high school	47 (60.53)	315 (40.85)	64 (41.61)	534 (34.90)
College and university	21 (27.63)	373 (48.51)	70 (45.64)	811 (53.06)
Post-graduate	0 (0.00)	45 (5.82)	6 (4.03)	116 (7.56)
Urbanization level				
Metropolitan	26 (33.77)	361 (46.84)	23 (15.28)	341 (22.30)
Satellite	22 (28.57)	214 (27.84)	50 (32.64)	533 (34.84)
Rural	29 (37.66)	195 (25.32)	80 (52.08)	656 (42.85)
Monthly-income-based premium (NTD)	17,748 ± 12,906	26,657 ±19,489	22,729 ± 14,613	27,272 ± 18,179
Dependent or <20,099	30 (38.96)	242 (31.47)	54 (35.29)	441 (28.80)
20,100∼23,099	26 (33.77)	206 (26.71)	54 (35.29)	405 (26.49)
24,000∼38,199	16 (20.78)	157 (20.35)	26 (16.99)	341 (22.27)
≥ NT$38,200	5 (6.49)	165 (21.47)	19 (12.42)	343 (22.44)
Low income family	≤3^a^	9 (1.17)	4 (2.61)	16 (0.35)
Married	61 (79.22)	697 (94.19)	136 (91.28)	4,132 (94.81)
***Co-morbidity during 3 years prior to *** ***the index date***				
Acute myocardial infarction	0 (0.00)	0 (0.00)	^c^	^c^
Ischemic heart disease	0 (0.00)	4 (0.56)	^c^	^c^
Coronary revascularization procedures	0 (0.00)	0 (0.00)	^c^	^c^
Non-traumatic hemorrhagic stroke	0 (0.00)	≤3^a^	^c^	^c^
Ischemic stroke	≤3^a^	3 (0.40)	^c^	^c^
Acute pancreatitis	≤3^a^	0 (0.00)	^c^	^c^
Urinary tract infection	39 (50.65)	242 (31.43)	^c^	^c^
Acute respiratory infections	66 (85.71)	705 (91.60)	^c^	^c^
Tuberculosis	0 (0.00)	3 (0.40)	^c^	^c^
Pneumonia	7 (9.09)	5 (0.65)	^c^	^c^
Sepsis	0 (0.00)	0 (0.00)	^c^	^c^
Acute renal failure	≤3^a^	0 (0.00)	^c^	^c^
Acute respiratory failure	≤3^a^	0 (0.00)	^c^	^c^
Hepatic failure	0 (0.00)	≤3^a^	^c^	^c^
Disseminated intravascular coagulopathy syndrome	0 (0.00)	0 (0.00)	^c^	^c^
Kidney disease	^b^	^b^	4 (2.61)	14 (0.89)
Cardiovascular disease	^b^	^b^	≤3^a^	10 (0.65)
Heart failure	^b^	^b^	0 (0.00)	3 (0.17)
Depression	^b^	^b^	6 (3.92)	36 (2.33)
Prior history of severe hypoglycemia			3 (2.00)	9 (0.61)
***Air pollutants***				
Particulate matter (PM_2.5_, µg/m^3^)	31.76 ± 13.38	31.10 ± 11.92	30.22 ± 10.28	31.38 ± 12.09
***Birth outcomes***				
Male offspring	27 (35.06)	357 (46.49)	80 (52.29)	727 (47.52)
Gestational weeks	35.57 ± 2.68	38.27 ± 1.48	36.99 ± 2.19	38.46 ± 1.46
Preterm (gestation <37 weeks)	42 (54.55)	67 (8.74)	51 (33.33)	102 (6.64)
Very preterm (gestation <34 weeks)	5 (6.49)	3 (0.38)	4 (2.61)	7 (0.44)
Birth weight (g)	2782.6 ± 750.3	3109.7 ± 443.2	3023.0 ± 684.9	3141.3 ± 440.0

Abbreviations: GDM, gestational diabetes mellitus; SD, standard deviation; NTD, New Taiwanese Dollar; 1 USD ≅ 27.5 NTD.

^a^The exact number below 3 is not specified, in accordance with Taiwanese privacy regulations.

^b^Co-morbidity not known to be risk factors for hyperglycemia emergency.

^c^Co-morbidity not known to be risk factors for severe hypoglycemia.

Associations of hyperglycemic emergency incidence with season and temperature are shown in Table 2. Compared to summer, both spring and winter were found to be associated with increased odds of hyperglycemic emergency after controlling for potential confounders, but the elevated covariate adjusted odds ratios (aOR) were compared to null statistically. In addition, per 1°C increase in mean daily average temperature was also not significantly associated with aOR of hyperglycemic emergency. On the other hand, compared with the controls, cases of severe hypoglycemia were more likely to occur in winter than in summer, with a significantly higher aOR of 1.74 (95% CI, 1.08–2.79) (Table 3). A 1 °C increase in mean daily average temperature showed little association with severe hypoglycemia, with an aOR of 0.99 (95% CI, 0.96–1.01) (Table 3).

**Table 2:** Associations of *hyperglycemic emergency* incidence with season and temperature.

**Season and temperature**	**Cases (%)**	**Controls (%)**	**Univariate analysis**		**Multivariate analysis**	

			**Crude OR (95% CI)**	***P* value**	**Adjusted OR (95% CI)^b^**	***P* value**
Season^a^						
Spring	19 (24.68)	188 (24.68)	1.25 (0.65–2.41)	0.5118	1.35 (0.69–2.67)	0.3815
Summer	18 (23.38)	220 (28.57)	1.00 (Ref.)	-	1.00 (Ref.)	-
Fall	16 (20.78)	198 (25.71)	0.99 (0.50–1.95)	0.9684	1.04 (0.52–2.10)	0.9083
Winter	24 (31.16)	167 (21.69)	1.79 (0.95–3.38)	0.0733	1.88 (0.97–3.64)	0.0598
Temperature						
Per 1 °C increase in mean daily average temperature			0.95 (0.91–0.99)	0.0346	0.97 (0.92–1.03)	0.3575

Abbreviations: CI, confidence interval; OR, odds ratio; Ref. Reference group.

^a^Spring: Mar. – May; Summer: Jun. – Aug.; Fall: Sep. – Nov.; Winter: Dec. – Feb.

^b^OR and 95% CI were estimated from conditional logistic regression model, with inclusion of maternal sociodemographic status, air pollution, selected co-morbidity including urinary tract infection, acute respiratory infections, and pneumonia in addition to season and temperature.

**Table 3:** Associations of *severe hypoglycemia* incidence with season and temperature.

**Season and temperature**	**Cases (%)**	**Controls (%)**	**Univariate analysis**		**Multivariate analysis**	

			**Crude OR (95% CI)**	***P* value**	**Adjusted OR (95% CI)^b^**	***P* value**
Season^a^						
Spring	34 (22.22)	395 (25.82)	1.07 (0.64–1.78)	0.7968	1.06 (0.64–1.76)	0.8213
Summer	32 (20.92)	400 (26.14)	1.00 (Ref.)	-	1.00 (Ref.)	-
Fall	39 (25.49)	390 (25.49)	1.24 (0.75–2.02)	0.4020	1.22 (0.75–2.00)	0.4280
Winter	48 (31.37)	345 (22.55)	1.74 (1.09–2.80)	0.0211	1.74 (1.08–2.79)	0.0222
Temperature						
Per 1 °C increase in mean daily average temperature			0.98 (0.95–1.01)	0.2458	0.99 (0.96–1.01)	0.3443

Abbreviations: CI, confidence interval; OR, odds ratio; Ref. Reference group.

^a^Spring: Mar. – May; Summer: Jun. – Aug.; Fall: Sep. – Nov.; Winter: Dec. – Feb.

^b^OR and 95% CI were estimated from conditional logistic regression model, with inclusion of maternal sociodemographic status, air pollution, selected co-morbidity including kidney disease, depression, and prior history of severe hypoglycemia in addition to season and temperature.

A subgroup analysis was conducted to further examine the associations of two acute glycemic complications with season and ambient temperature in pregnancies with T1DM, T2DM, and GDM, separately. We were unable to estimate the regression coefficients for the analysis of pregnancies with T2DM due to limited sample size. Season was not significantly associated with hyperglycemia emergency in pregnancies with T1DM or GDM. On the other hand, fall (1.90, 95% CI, 1.02–3.54) and winter (3.90, 95% CI, 1.60–9.51) were found to be associated with significantly increased aORs of severe hypoglycemia in pregnancies with T1DM. Although less evident, fall (1.99, 95% CI, 0.50–7.92) and winter (2.79, 95% CI, 0.95–8.19) were also found to be associated with increased aORs of severe hypoglycemia in pregnancies with GDM. Ambient temperature was, on the other hand, not found to be significantly associated with risks of hyperglycemia emergency or severe hypoglycemia in any subgroup (Supplementary Table 3).

## Discussion

### Main findings

While there was no association between ambient temperature and acute glycemic complications, our study showed elevated risks of severe hypoglycemia, but not hyperglycemic emergency in diabetic pregnancies during winter season, which was independent of ambient temperature. The association between seasonality and severe hypoglycemia tended to be more evident in pregnancies with T1DM. These findings call for the attention of clinicians and pregnant women with diabetes, especially in the context of documented global rise in the prevalence of pre-pregnancy diabetes and GDM [32].

### Strengths and limitations

To the best of our knowledge, this is the first epidemiological study investigating the associations of seasonality and ambient temperature with acute glycemic complications in Southeast Asia, a region that exhibits the world’s highest prevalence of diabetes in pregnancy in 2019 [33]. Other methodological strengths included a population-based nested case-control design that greatly minimizes the potential for selection bias. Additionally, with such a large sample size, this study has adequate statistical power to detect a moderate association between seasonality and acute glycemic complications, a very rare event occurring during pregnancy. Besides, simultaneous including of season and ambient temperature in the model help make interpretation of study findings.

Mounting evidence has shown that sustained cold temperature may expand the volume of brown adipose tissue, which in turn affect insulin sensitivity [34–36]. Previous experimental studies also found an effect of ambient temperature on redistribution of blood flow [37]. Unlike the above direct biological responses to ambient temperature, season is usually associated with a broader context. Winter is usually associated with a short daytime and poor road conditions, thus affecting the access to and continuity of health care for glucose control [38]. Evidence also indicates that winter season and cold weather are associated with reduced physical activities and high food intake [39]. Such seasonal fluctuations in lifestyle may reflect a link between feeding-related hormones (e.g., ghrelin and leptin) and seasonal changes in the circulating levels of glucocorticoids [40].

Despite the above strengths, several methodological limitations should be acknowledged. First, this study relies solely on claims and registration data that precluded mild hypoglycemia that requires no medical treatment. Exclusion of mild hypoglycemia might result in disease misclassification in which some cases might have dropped and the control group might have comprised of some cases with mild hypoglycemia. Besides, Birth Notification includes only pregnancies that end up with live births or therapeutic abortions (normally >=20 weeks of gestation) and the potential for selection bias might arise from missing some cases of early spontaneous abortions. Second, we used maternal residential information at the time of end-of-pregnancy, which might have entailed potential exposure (temperature) misclassification due to maternal mobility during pregnancy. Third, we assumed a 30-day exposure window biologically relevant to the occurrence of acute glycemic events. If temperature has a shorter effect, our current analysis may result in the exposure misclassification, leading to an underestimation of the true association. Similar problems can be applied to certain covariates such as PM_2.5_. Fourth, we did not have specific information on lifestyle, an information unavailable from electronic medical records, which makes it difficult to specify the lifestyle most relevant to the increased risk of severe hypoglycemia. Fifth, we used 10 matched controls per case, which is subject to overpower albeit a large number of controls did not incur extra cost in such a study based on existing datasets. Lastly, treatment modalities and their duration exert some influence on the frequency and severity of acute glycemic complications in patients with T1DM and T2DM [41]. A recent study also found that certain oral anti-glucose agents such as glyburide are as safe and effective as insulin but not for the high rates of hypoglycemia in women with GDM [42]. Some non-traditional risk factors including cognitive and physical functioning are also strongly associated with increased cases of severe hypoglycemia [43]. Although medication use and treatment modalities were not considered in our analysis, these factors are unlikely to pose decisive influence on the results because selection of therapies for diabetes in pregnant women is unlikely to be associated with season. In addition, the prevalence of poor cognitive and physical functioning is considered very low in young women of reproductive ages.

### Interpretation

Our study revealed significantly elevated OR of severe hypoglycemia in winter season among pregnant women with diabetes, and such association was independent of ambient temperature. The findings are consistent with some previous studies [7, 8, 44], but disagreed with some others [45, 46]. A higher prevalence of severe hypoglycemia noted in our study can have a multifaceted interpretation. First, although the prescriptions to glucose-lowering medications is not likely to present obvious seasonal variation, a Japanese study demonstrated that severe glucose-lowering drug-induced hypoglycemia occurred more frequently in the cold season than in the warm season, and was associated with an inflammatory state in patients treated with sulfonylurea [44]. Second, a seasonal variation regarding hospitalization was observed for severe hypoglycemia among a sample of Japanese patients with type 2 diabetes, with a higher incidence in the cold season than the warm season, and anorexia related to infections [47]. Because people living with diabetes are vulnerable to infection which is more common in cold seasons than in warm ones, pregnant women with pre-pregnancy diabetes might be at higher risk of infection, leading to an increased risk of severe hypoglycemia in winter. Nonetheless, comparisons of results between previous studies and ours may not be so straightforward because previous studies did not specifically focus on pregnant women with diabetes, which could be clinically and biologically different from the general diabetes population.

A recent Taiwanese study also reported that patients with type 2 diabetes in Taiwan are more susceptible to hypoglycemia during the winter season [8]. But another Taiwanese study reported that patients with type 2 diabetes were at higher risk of HbA1c >7% in the winter than in the summer, however, hypoglycemia was not examined in the study [48]. While there was a clustering of hypoglycemia hospitalization among type 1 diabetes patients in March, a Canadian observational study did not show variation for hypoglycemia hospitalizations by season [49]. Although our study is unable to explain the true mechanisms of the association between winter season and severe hypoglycemia, the observed associations are more likely due to winter season related lifestyle or activities rather than ambient temperature itself.

The link between diabetes and poor pregnancy outcomes is well established. Poor control of diabetes during pregnancy causes serious complications for women and increases the chances for various health problems in offspring, including congenital anomalies, fetal overgrowth, and stillbirth [50]. The disease burden associated with pregnancy with diabetes is particularly noteworthy because of the worldwide increase in the prevalence of T2DM, which has apparently similar or even worse outcomes to T1DM [51]. In addition, the prevalence of GDM, one of the most common pregnant complications, has also increased by more than 30% within one or two decades in a number of countries including developing countries, thus creating an emerging worldwide epidemic [52]. Given the increase in the prevalence of diabetes in pregnancy, appropriate strategies and preventive measures should be undertaken to mitigate the potential adverse effect from cold season on acute glycemic complications in pregnant women with diabetes.

## Conclusions

In conclusion, a moderately but significantly elevated risk of severe hypoglycemia was found in pregnant women with diabetes during winter season, which is independent of ambient temperature and other known clinical risk factors for severe hypoglycemia. Future studies are needed to find out the specific lifestyle change during winter season most relevant to the elevated risk. Both clinicians and pregnant mothers with diabetes should be aware of the potential influences of seasonality on acute glycemia complications.

## Abbreviations

DKA: diabetic ketoacidosis
HHS: hyperosmolar hyperglycemic state
T1DM: type 1 diabetes mellitus
T2DM: type 2 diabetes mellitus
NHI: National Health Insurance
NHIA: National Health Insurance Administration
GDM: gestational diabetes
OR: odds ratio
CI: confidence interval
SD: standard deviation
NTD: New Taiwan Dollar

## Supplementary information

**Additional file 1:** **Supplementary Table 1.** Sociodemographic characteristics and season of and of all pregnancies registered in Birth Notification from 2009 to 2014 in Taiwan, *n* = 1,212,569 pregnancies by (938,992 mothers). **Supplementary Table 2.** International Classification of Diseases, 9th Revision, Clinical Modification (ICD-9-CM) codes used to identify comorbidities. **Supplementary Table 3.** Subgroup analyses of odds ratios for hyperglycemia emergency and severe hypoglycemia in relation to seasonality and ambient temperature.

## Declarations

### Availability of data and material

Data management and all analyses were performed onsite at the Health and Welfare Data Science Center of the Taiwan Ministry of Health and Welfare. Data is not available to the public and data sharing is prohibited under the current government regulations.
