# Supplementary material for: Associations of hyperglycemic emergency and severe hypoglycemia incidences with seasonality and ambient temperature among pregnant women with diabetes: a nested case-control study in Taiwan
Source: Environ Health Prev Med. 2022 Mar 12;27:11. doi: 10.1265/ehpm.22-00003 (PMC9093638; doi:10.1265/ehpm.22-00003)
Supplement: Supplementary file 1 — Additional file 1: Supplementary Table 1. Sociodemographic characteristics and season of and of all pregnancies registered in Birth Notification from 2009 to 2014 in Taiwan, n = 1,212,569 pregnancies by (938,992 mothers). Supplementary Table 2. International Classification of Diseases, 9th Revision, Clinical Modification (ICD-9-CM) codes used to identify comorbidities. Supplementary Table 3. Subgroup analyses of odds ratios for hyperglycemia emergency and severe hypoglycemia in relation to seasonality and ambient temperature. [file ehpm-27-011-s001.docx]

Supplementary Table 1. Sociodemographic characteristics and season of and of all pregnancies registered in Birth Notification from 2009 to 2014 in Taiwan, *n*=1,212,569 pregnancies by (938,992 mothers)

| Variables | *n* (%) ^a^ |
| --- | --- |
| *Maternal sociodemographic status* |  |
| Maternal age at index date (years), n (%) |  |
| 15-24 | 134,434 (11.19) |
| 25-29 | 345,771 (28.79) |
| 30-34 | 489,758 (40.78) |
| 35-50 | 230,965 (19.23) |
| Aboriginal people, n (%) | 38,638 (3.42) |
| Born in Taiwan, n (%) | 1,120,448 (92.40) |
| Education, n (%) |  |
| Junior high school or below | 67,818 (5.59) |
| Senior high school | 418,105 (34.48) |
| College and university | 639,628 (52.75) |
| Post-graduate | 87,018 (7.18) |
| Urbanization level, n (%) |  |
| Metropolitan | 408,660 (33.70) |
| Satellite | 443,158 (36.55) |
| Rural | 360,751 (29.75) |
| Monthly-income-based premium (NTD), n (%) |  |
| Dependent or <20,099 | 360,043 (29.69) |
| 20,100∼23,099 | 303,274 (25.01) |
| 24,000∼38,199 | 276,675 (22.82) |
| ≥ NT$38,200 | 272,577 (22.48) |
| Low income family, n (%) | 7,644 (0.63) |
| Married, n (%) | 1,053,975 (86.92) |
| *Season of delivery*, n (%) |  |
| Spring | 298,883 (24.65) |
| Summer | 311,251 (25.67) |
| Fall | 326,832 (26.95) |
| Winter | 275,603 (22.73) |

NTD, New Taiwan Dollar, 1 USD ≅ 27.5 NTD

^a^ Inconsistency between total population and population summed for maternal age was due to missing information

Supplementary Table 2. International Classification of Diseases, 9th Revision, Clinical Modification (ICD-9-CM) codes used to identify comorbidities.

| Comorbidities | ICD-9-CM |
| --- | --- |
| Acute myocardial infarction | 410 |
| Ischemic heart disease | 410-414 |
| Coronary revascularization procedures | 36.0, 36.01, 36.02, 36.05, 36.06, 36.1, 36.10-36.19 |
| Non-traumatic hemorrhagic stroke | 430-432 |
| Ischemic stroke | 433-438 |
| Acute pancreatitis | 577.0 |
| Urinary tract infection | 590.1, 595.0, 595.9, 599.0 |
| Acute respiratory infections | 460-466 |
| Pneumonia and influenza | 480-488 |
| Tuberculosis | 010-018 |
| Sepsis | 995.92 |
| Acute renal failure | 584.5-584.9 |
| Acute respiratory failure | 518.81 |
| Hepatic failure | 570 |
| Disseminated intravascular coagulopathy syndrome | 286.6 |
| Kidney disease | 580-589 |
| Cardiovascular disease |  |
| Coronary artery disease | 410, 411, 413, 428; A-code: A291, A292, A293, A299 |
| Cerebral vascular attack | 430-436; A-code: A291, A292, A293, A299 |
| Heart failure | 428 |
| Depression | 296, 309, 311 |

Supplementary Table 3. Subgroup analyses of odds ratios for hyperglycemia emergency and severe hypoglycemia in relation to seasonality and ambient temperature

| Season and ambient temperature ^a^ | Hyperglycemia emergency | | | |  | Severe hypoglycemia | | | |
| --- | --- | --- | --- | --- | --- | --- | --- | --- | --- |
|  | Case (%) | Control (%) | Adjusted OR  (95% CI) ^b^ | P value |  | Case (%) | Control (%) | Adjusted  OR (95% CI) ^c^ | P value |
| Pre-pregnancy type 1 diabetes | *n*=53 | *n*=530 |  |  |  | *n*=69 | *n*=690 |  |  |
| Season |  |  |  |  |  |  |  |  |  |
| Spring | 13 | 127 | 1.07 (0.36-3.18) | 0.8968 |  | 17 | 170 | 1.87 (0.89-3.93) | 0.2118 |
| Summer | 12 | 140 | 1.00 (Ref.) | - |  | 14 | 199 | 1.00 (Ref.) | - |
| Fall | 12 | 146 | 0.84 (0.30-2.35) | 0.8020 |  | 17 | 173 | 1.90 (1.02-3.54) | 0.0304 |
| Winter | 16 | 117 | 1.35 (0.38-4.80) | 0.7211 |  | 21 | 148 | 3.90 (1.60-9.51) | <0.001 |
| Ambient temperature ^d^ |  |  | 0.95 (0.76-1.20) | 0.5024 |  |  |  | 0.98 (0.87-1.10) | 0.5812 |
| Gestational diabetes mellitus | *n*=21 | *n*=210 |  |  |  | *n*=71 | *n*=710 |  |  |
| Season |  |  |  |  |  |  |  |  |  |
| Spring | 5 | 47 | 1.12 (0.32-3.92) | 0.8644 |  | 16 | 159 | 2.25 (0.65-7.79) | 0.2600 |
| Summer | 6 | 60 | 1.00 (Ref.) | - |  | 16 | 201 | 1.00 (Ref.) | - |
| Fall | 4 | 61 | 0.68 (0.31-1.49) | 0.7322 |  | 19 | 195 | 1.99 (0.50-7.92) | 0.3684 |
| Winter | 6 | 42 | 1.36 (0.36-5.14) | 0.6514 |  | 20 | 155 | 2.79 (0.95-8.19) | 0.0733 |
| Ambient temperature  ^d^ |  |  | 1.00 (0.99-1.02) | 0.9864 |  |  |  | 1.01 (0.98-1.04) | 0.8194 |

^a^ Subgroup analysis was not performed for pre-pregnancy type 2 diabetes due to limited sample size of pre-pregnancy type 2 diabetes cases.

^b^ OR and 95% CI were estimated from conditional logistic regression model, with adjustment for maternal sociodemographic status, air pollution, apparent temperature, and selected co-morbidity including urinary tract infection, acute respiratory infections, and pneumonia.

^c^ OR and 95% CI were estimated from conditional logistic regression model, with adjustment for maternal sociodemographic status, air pollution, apparent temperature, and selected co-morbidity including kidney disease, depression, and prior history of severe hypoglycemia.

^d^ Per 1°C increase in mean daily average temperature
